# The Pharmacologic Treatment of Stuttering and Its Neuropharmacologic Basis

**DOI:** 10.3389/fnins.2020.00158

**Published:** 2020-03-27

**Authors:** Gerald A. Maguire, Diem L. Nguyen, Kevin C. Simonson, Troy L. Kurz

**Affiliations:** Department of Psychiatry and Neuroscience, School of Medicine, University of California, Riverside, Riverside, CA, United States

**Keywords:** stuttering, medication, pharmacologic, risperidone, ecopipam, olanzapine, aripiprazole

## Abstract

Stuttering is a DSM V psychiatric condition for which there are no FDA-approved medications for treatment. A growing body of evidence suggests that dopamine antagonist medications are effective in reducing the severity of stuttering symptoms. Stuttering shares many similarities to Tourette’s Syndrome in that both begin in childhood, follow a similar male to female ratio of 4:1, respond to dopamine antagonists, and symptomatically worsen with dopamine agonists. In recent years, advances in the neurophysiology of stuttering have helped further guide pharmacological treatment. A newer medication with a novel mechanism of action, selective D1 antagonism, is currently being investigated in FDA trials for the treatment of stuttering. D1 antagonists possess different side-effect profiles than D2 antagonist medications and may provide a unique option for those who stutter. In addition, VMAT-2 inhibitors alter dopamine transmission in a unique mechanism of action that offers a promising treatment avenue in stuttering. This review seeks to highlight the different treatment options to help guide the practicing clinician in the treatment of stuttering.

## Introduction

Childhood-onset fluency disorder (stuttering) is defined by the American Psychiatric Association (APA) Diagnostic and Statistical Manual (DSM), fifth edition (V), as a disturbance in the normal fluency and time pattern of speech that is inappropriate for the individual’s age and persists over time. Repetitions, prolongations, broken words, blocking, circumlocutions, and excess physical tension characterize these disturbances. Motor movements (e.g., eye blinks, tics, tremors, head jerking, breathing movements) may accompany stuttering. The extent of these disturbances varies situationally and can be associated with fearful anticipation of stuttering, which exacerbates dysfluency. The resulting anxiety, embarrassment, insecurity, stress, shame, and bullying can cause limitations in effective social participation and academic or occupational achievement. For many individuals, avoidance and social anxiety are often the disabling features of this condition.

Previously classified as “stuttering” and listed as an Axis 1 disorder in the DSM IV, the APA modified the classification and description of stuttering for the DSM V published in 2013. This included changing the diagnostic label from “stuttering” to “childhood-onset fluency disorder,” removing the interjection criteria, inclusion of avoidance/anxiety criteria concerning speaking situations, and further distinguishing adult-onset stuttering from childhood-onset fluency disorder (American Psychiatric Association Dsm-5 Task Force, 2013).

Dysfluency usually starts gradually, affecting single words, but becomes more frequent and interferes with complete phrases as the disorder progresses. Age of developmental stuttering onset ranges from 2 to 7 years, with 80–90% of affected individuals showing symptoms by age 6. This chronic speech motor disorder affects approximately 5% of children; however, recent data suggest a lifetime incidence upward of 10%, with most incidents occurring in children (Yairi and Ambrose, 2013). Longitudinal research shows that 65–85% of children recover from dysfluency by age 16, leading to prevalence of less than 1% in adults (Andrews et al., 1983; Andrews and Craig, 1988).

Stuttering meets criteria as a disorder when it causes functional impairments, and early intervention has the best long-term outcomes. Stuttering has a high association with other DSM V diagnoses, likely secondary to the cumulative negative impact of stuttering (Craig and Tran, 2014; Iverach and Rapee, 2014). Evidence suggests that individuals with stuttering have increased risk of developing social anxiety, which often begins in adolescence and continues throughout adulthood (Smith et al., 2014). Adults who stutter have a twofold increase in mood disorders and threefold increase in personality disorders when compared to controls (Iverach et al., 2009a). Stuttering in adults has been associated with lower quality of life, occupation and educational barriers, and difficulties with access to high-quality treatment plans (Orton, 1927; Koedoot et al., 2011). There remains no medication with FDA approval for the treatment of stuttering. Continued research into stuttering will allow clinicians to understand its causes, pathophysiology, and treatment in order to assure the most appropriate care.

## Etiology

Stuttering has occurred in every culture throughout recorded history, yet to this day the exact cause remains unknown. Historically, stuttering was considered secondary to physical abnormalities of the larynx and tongue; however, surgical and chemical treatments focused on these anatomical areas did not improve symptoms. It wasn’t until the early 20th century that Orton and Travis conceptualized stuttering as a brain abnormality. They postulated that stuttering may arise from abnormal cerebral activity, leading to new theories regarding the etiology of stuttering. Psychoanalytic theory then attempted to explain stuttering as unconscious neurotic need fulfillment with unresolved oral conflict during one’s early parent–child interactions. Unfortunately, this furthered the stigma of stuttering. Stuttering is now viewed as a neurologic disorder brought on by incomplete dominance of the primary speech centers in the brain with a multifactorial etiology (Travis, 1931).

Genetics are thought to be involved in many cases of stuttering, with twin and family studies suggesting genetics account for 50–80% of stuttering, while fraternal studies suggest a 19% genetic correlation (Yairi and Ambrose, 2013). Twin studies indicate that monozygotic twins consistently display higher concordance for stuttering than dizygotic twins and estimated heritability has shown to exceed 0.80 in some studies (Ooki, 2005; Fagnani et al., 2011; Rautakoski et al., 2012). Other studies indicate a risk of stuttering to be three times higher in those with first-degree biological relatives compared to the general population.

Several studies focused on the genetic basis for stuttering have identified a single process of intracellular trafficking as the cellular defect for the disorder. These studies provide evidence for a strong genetic factor pertaining to stuttering with linkage associated to genes on chromosomes 9, 10, 12, 13, and 18. However, the results do not conclusively identify any specific genes that can contribute to the development of stuttering within the population at large (Wittke-Thompson et al., 2007; Lan et al., 2009; Kang et al., 2010). Newer studies have demonstrated an association between dopaminergic genes (SLC6A3 and DRD2) and stuttering, further supporting the dopamine theories of stuttering (Montag et al., 2012; Chen et al., 2014).

Stuttering shares many similarities with Tourette’s Syndrome – both start in childhood, have 4:1 male to female ratio, have a waxing and waning course, are made worse with anxiety, are associated with tic motions, have brain abnormalities localized to the basal ganglia, have symptoms worsened by dopamine agonists, and symptoms improved with dopamine antagonists.

A recent case report also suggests that certain cases of stuttering could be due to pediatric autoimmune disorders associated with streptococcus infections (PANDAS) (Maguire G. A. et al., 2010). PANDAS has more established etiologic mechanisms in Tourette syndrome and obsessive–compulsive disorders. Some case studies hypothesize that stuttering occurs when antibodies directed against streptococcal infection cross-react and attack the developing basal ganglia.

Finally, there are rare cases of acquired stuttering that begin in adulthood that are related to iatrogenic causes, including medications and brain trauma (Ludlow and Dooman, 1992).

## Imaging

Brain imaging and neurophysiological tools have begun to elucidate the dysfunctional neural dynamics in developmental stuttering. Overall, it appears that people who stutter show decreased activity in brain areas associated with language processing (left-sided cortical speech sites) and dysfunctional activity in areas associated with the timing and coordination of motor function (basal ganglia) (Speech production, 1997; Chang and Zhu, 2013; Neef et al., 2016). Spontaneous stuttering is hypothesized to be secondary to a defect in the inner subcortical speech loop, including the striatum of the basal ganglia. Abnormally low function of the left striatum can lead to low activity in left-cortical speech areas. Induced fluency through techniques such as chorus reading shows increased activity of left-hemispheric speech areas equal to normal controls. Clinicians theorized that induced fluency activates the outer cortical speech loops, bypassing the inactive striatum in the inner speech loop (Chang et al., 2016). Furthermore, the low function of the striatum in stuttering is associated with an overactive presynaptic dopamine system disrupting the selection, initiation, and execution of motor sequences necessary for fluent speech production (Wu et al., 1997).

Structural neuroimaging has mapped morphological changes in people who stutter, leading to the hypothesis that the main deficit in stuttering is an impaired feedforward control system in the left hemisphere, forcing a compensatory overreliance on the feedback control system of the right hemisphere over time (Chang et al., 2019). Children who stutter are noted to have smaller volume and decreased white matter integrity/connectivity in tracts of the left hemisphere, compared to fluent peers (Chow and Chang, 2017). Adults who stutter are noted to have larger volume and increased white matter integrity in tracts of the right hemisphere, with increased right-frontal structural connectivity negatively correlated with stuttering severity (Jancke et al., 2004; Neef et al., 2018). Furthermore, neural oscillations reflecting rhythmic fluctuations of neuron excitability appear overly exaggerated in adults who stutter, with increased beta desynchronization and synchronization during speech preparation and execution compared to fluent controls (Mersov et al., 2018). Gender differences in the prevalence of stuttering have also been noted in neuroimaging studies, with decreased connectivity between the left motor to left pars opercularis in boys but not girls who stutter (Chang and Zhu, 2013). It is speculated that girls with intact connectivity of this region are more likely to spontaneously recover from stuttering, perhaps explaining the skewed sex ratio in persistent stuttering onto adulthood (Chang and Zhu, 2013).

Other brain imaging studies measuring glucose metabolism (FDG-PET) showed an association with abnormally low activity of speech cortical areas (Broca’s and Wernicke’s) and low activity of the striatum in stuttering subjects. Interestingly, when fluency was induced in these subjects, cortical speech areas increased to normal or high-normal activity, but striatal activity remained low (Wu et al., 1997). PET scans measuring 6-FDOPA uptake as a marker of presynaptic dopamine activity in stuttering subjects also illustrated almost a threefold increase in 6-FDOPA uptake compared to normal controls in the right ventral medial prefrontal cortex and left caudate tail (Wu et al., 1997). FDOPA uptake was increased by >100% in limbic structures, including the deep orbital cortex, insular cortex, and extended amygdala, suggesting an overactive mesocortical dopamine tract in those who stutter (Wu et al., 1997).

## Pharmacologic Treatment of Stuttering

Currently there is no FDA-approved medication for the treatment of stuttering. Medications with dopamine-blocking activity have shown the most efficacy; however, they can be limited by their respective side-effect profiles. Other agents have been tried in the past with limited efficacy, but newer medications with novel mechanisms are showing promise in the pharmacologic treatment of stuttering.

Early research in 1980 illustrated that a first-generation dopamine-blocking antipsychotic, haloperidol, improved fluency by increasing brain activity in speech areas (Wood et al., 1980). Unfortunately, haloperidol has low tolerability and poor long-term compliance due to disabling side-effects (e.g., dysphoria, sexual dysfunction, extrapyramidal symptoms, and tardive dyskinesia) (Rosenberger et al., 1976). Nevertheless, haloperidol research led to further brain imaging studies (SPECT), which revealed that stuttering was associated with abnormally low brain activity in left-sided speech cortical areas (Pool et al., 1991).

It is postulated that elevated dopamine levels are associated with stuttering and lower activity of the striatum, supported by a 1997 study showing significantly higher 6-FDOPA uptake in the ventral limbic cortical and subcortical regions leading to an overactive presynaptic dopamine system (Wu et al., 1997). It is also known that atypical antipsychotic medications such as olanzapine and risperidone block dopamine at the D2 receptor, thus leading to increased activity of the striatum and improved fluency (Maguire et al., 2002). Furthermore, dopamine agonists, medications that enhance the activity of dopamine (the opposite of dopamine blocking), such as L-dopa, worsen the symptoms of stuttering (Burd and Kerbeshian, 1991).

Pimozide, another dopamine blocking medication similar to haloperidol, showed positive clinical responses but can be associated with treatment-limiting side-effects (e.g., EPS, TD, dysphoria, prolactin elevation, and cardiac conduction concerns) (Bloch et al., 1997). In contrast, paroxetine, an antidepressant medication that decreases the reuptake of serotonin (SSRI), exhibited no clinical response in stuttering (Stager et al., 2005). Like SSRIs, tricyclic antidepressants have shown little benefit for the treatment of stuttering. A comparison of clomipramine and desipramine showed minimal short-term improvements in fluency and self-reported speaking avoidance, with clomipramine showing superior improvement then desipramine on self-reported scales of fluency in another analysis (Gordon et al., 1995; Stager et al., 1995).

Newer, second-generation dopamine-blocking medications such as risperidone and olanzapine have a lower risk of motor system side-effects (e.g., tardive dyskinesia) and are generally better tolerated than first-generation dopamine-blocking medications like haloperidol. Risperidone is associated with increased activity in the striatum and cortical speech areas and significantly decreased overall stuttering severity at doses between 0.5 and 2 mg/day (Maguire et al., 2000a). While risperidone is generally well tolerated, it can increase blood levels of the hormone prolactin, leading to potentially concerning side-effects including sexual dysfunction, galactorrhea, amenorrhea, and dysphoria. In a case series of risperidone treatment by Maguire et al., the mean change score in the stuttering frequency (%SS) of the risperidone group was –4.83 (SD = 3.72) compared to placebo –2.11 (SD = 2.66), with a Cohen’s *d* of 0.84 indicating a large effect size. An NNT cannot be calculated based on the statistical analysis of the study utilizing mean reductions (Maguire et al., 2000a). Risperidone was also associated via FDG PET imaging to be associated with increased metabolism of left striatal function compared to patients treated with placebo (Maguire et al., 2000b).

Further research shows that olanzapine possesses a different tolerability profile than risperidone (fewer motor symptom side-effects, sexual dysfunction, and prolactin elevation), but does have a greater propensity for significant weight gain (Tran et al., 1997). While olanzapine at doses between 2.5 and 5 mg has been more effective than a placebo at reducing stuttering, it is also correlated with an average of 4 kg weight gain (Maguire et al., 2004). In a double-blind, placebo-controlled trial by Maguire et al. (2004) olanzapine was statistically superior to placebo in improving symptoms according to different rating systems of stuttering severity. The percent reduction on the subjective stuttering scale 22% on active medication and <1% on placebo. A more recent 2013 study compared the effects of olanzapine versus haloperidol in controlling the signs and symptoms of stuttering, with results showing olanzapine reduced the severity of stuttering more than haloperidol and may be the recommended first-choice medication for individuals who stutter (Shaygannejad et al., 2013). Olanzapine has also been noted to induce down-regulation of postsynaptic GABA-A receptors, suggesting that directly acting GABA-A agonists or partial agonists may have benefit in the treatment of stuttering (Farnbach Pralong et al., 1998).

A recent case report demonstrated ziprasidone to be an effective and well tolerated medication for the treatment of stuttering and may be considered as an alternative atypical antipsychotic (Munjal et al., 2018). Additional newer dopamine antagonist medications include asenapine, which has less association with significant weight gain or glucose/lipid increases compared to olanzapine. Asenapine utilizes sublingual administration, which is absorbed more quickly. Asenapine, in a limited open-label trial for stuttering, indicated improved fluency on well-tolerated doses of 5–10 mg (Maguire et al., 2011).

Aripiprazole is a unique medication that acts as a partial agonist of D2 and 5HT1a receptors. It is FDA-approved for Tourette’s in children and adults. There are published case reports examining the safety and efficacy in stuttering (at dosages of 15 mg per day) for adults and adolescents (Hoang et al., 2016). However, akathisia is a side-effect that can limit aripiprazole’s utility in stuttering. There is a generic version available that may make it more cost-effective than other new medications (Tran et al., 2008).

Lurasidone is another newer dopamine antagonist with a unique pharmacologic profile. It is approved in children/adolescents for schizophrenia (13–17 years old) and bipolar depression (10–17 years old). A small open-label study of lurasidone in patients with stuttering showed improvement in the Subjective Screening of Stuttering (SSS) Scale. Improvement was also seen in subjective symptoms and the Clinical Global Impression Scale. Advantages include less sedation and lower risk of metabolic side effects (including weight gain and lipid elevations) (Charoensook and Maguire, 2017).

Numerous medications for stuttering are have been studied, but until recently only those with dopamine blocking activity have confirmed efficacy. Pagoclone, a selective GABA-A partial agonist, was theorized to have downstream effects on dopamine; however, it showed limited efficacy in the largest pharmacologic trial of stuttering ever conducted. Pagoclone showed strong placebo response in the trial and was likely under-dosed. It is possible that pagoclone decreased stuttering by lowering social anxiety levels, which can make stuttering worse. There has been no further development of this compound (Maguire G. et al., 2010).

Clonidine is an alpha receptor agonist, shown to be effective in controlling signs and symptoms of Tourette’s Syndrome. It was thus hypothesized that clonidine may be effective for stuttering; however, a well-designed study failed to show any difference between clonidine and placebo for objective measures of stuttering as well as parent and teacher ratings (Althaus et al., 1995).

Calcium channel blockers such as verapamil and nimodipine have also shown limited efficacy in stuttering in separate studies (Brumfitt and Peake, 1988; Brady et al., 1990). GABA receptor agents have also been investigated due to their known anxiolytic effects, including benzodiazepines and barbiturates. They were shown to reduce anxiety short term; however, they have not shown to improve fluency in stuttering and did not demonstrate any benefits compared to placebo (Sedlackova, 1970; Novak, 1975; Brady, 1991).

Ecopipam has a unique pharmacologic mechanism in its action as a D1 antagonist. This is different from other dopamine antagonists, which mostly act on the D2 receptor. Also, unlike other dopamine antagonists, ecopipam is an investigational drug not FDA approved for any other conditions, but it has shown efficacy in adolescent Tourette’s. An open-label study of ecopipam in adults demonstrated no reports of parkinsonian-like EPS typically seen with D2 antagonists. In addition, ecopipam had no reported weight gain; in fact, subjects experienced weight loss. Ecopipam has been studied for stuttering in adults in an open-label single-case experimental design funded by philanthropy. The results revealed that Ecopipam significantly improved stuttering symptoms on objective and subjective scales including the Overall Assessment of the Speaker’s Experience of Stuttering (OASES), which measures the impact of stuttering on a person’s life. Ecopipam was also well-tolerated, so further research is warranted. Ecopipam was also associated in this short-term study with improved quality of life in individuals who stutter (Maguire et al., 2019).

Another category of new medications under review is vesicular monoamine transporter 2 (VMAT2) inhibitors. Valbenazine and deutetrabenazine decrease the synthesis of dopamine through inhibition of VMAT2, a transport protein that packages dopamine into synaptic vesicles for release within the central nervous system. VMAT inhibitors have shown efficacy in Tourette’s, Tardive Dyskinesia, and abnormal movements associated with Huntington’s. One drawback is that VMAT2 inhibition is non-selective for monoamines and decreased serotonin could precipitate symptoms of depression; however, newer forms appear to lower that risk.

## Non-Pharmacologic Treatment of Stuttering

Non-pharmacologic treatments for stuttering range from non-invasive to maximally invasive approaches. Most established is speech therapy, which is supported by a large body of literature and has been proven to target different physiological centers of the brain.

Various speech and behavioral therapies for stuttering have shown limited significant differences in controlled clinical trials across time with higher relapse rates and negative effects on speech naturalness (Novelli, 2018). As stuttering tractability decreases during the school-age years, the Lidcombe program was developed for preschool children based on operant principles, with verbal contingencies for stuttering administered by the parents (de Sonneville-Koedoot et al., 2015). In a large randomized controlled trial presented by Sonneville-Koedoot et al., direct treatment with the Lidcombe program versus indirect treatment of reducing communicative pressures showed greater decline in stuttering at 3 months with the Lidcombe program, but comparable outcomes in stuttering frequency at 18 months. There were no significant differences in treatment approaches (de Sonneville-Koedoot et al., 2015). In children aged 9–14, Craig et al. (1996) showed that therapeutic treatment with intensive smooth speech, intensive electromyography feedback, and home-based smooth speech showed decreased stuttering frequency of 85–90% across all assessment contexts, regardless of treatment modality. Intensive smooth speech showed more immediate improvement (<1% SS); however, participants showed better long-term success with the EMG and home-based smooth speech 1 year post-treatment. There were no statistically significant differences between the three treatment groups when measuring stuttering frequency across time (Craig et al., 1996).

Using the anomalies in brain morphology and activations during fluent speech production, recent data postulates a model of spontaneous recovery versus therapy-induced assisted recovery. Developmental stuttering is associated with reduced cortical gray matter of the left inferior frontal region and with a secondary basal ganglia dysfunction independent from recovery (Kell et al., 2009). An fMRI study by Neumann et al. illustrated that this hypoactivation can be normalized after therapy-induced modification of speech melody and frequency, even 1 year post-therapy (Neumann et al., 2018). Fluency-induced therapies are associated with a shift of over-activations to the left hemisphere to normalize the merging auditory feedback and motor program (Kell et al., 2009). Auditory feedback controls the rhythm of articulation and dysfluency can be corrected by temporal auditory feedback manipulation. Furthermore, therapy has been shown to decrease the compensatory over-activation in the right lateral prefrontal and parietal regions involving attentional and executive control. Yet, while fluency-inducing therapies can assist in restoring a left dominant network for speech production, this effect requires continued maintenance through refresher therapies (Kell et al., 2009; Neumann et al., 2018).

Furthermore, as a large percentage of early spontaneous recoveries occur around age 3, Alm hypothesized an association with spontaneous early recovery and the natural phase of basal ganglia development. There is a significant peak in the D2 dopamine receptors in the basal ganglia occurring at age 2.5–3 (Alm, 2004). The dual premotor systems model of stuttering emphasized the basal ganglia as part of the larger medial system that is dominant during spontaneous, automatic speech, especially in speech conveying thought and emotions. Behavioral therapy modalities, such as metronome-timed speech, unison reading, accent imitation, and role-play, are believed to bypass control from the medial to the lateral system (consisting of the lateral premotor cortex and cerebellum) to produce attentional, controlled speech based on auditory and somatosensory feedback (Alm, 2004).

Other non-pharmacological therapeutic interventions may be beneficial, including different forms of psychotherapy such as cognitive behavioral therapy (CBT). In the management of chronic stuttering, the importance of social anxiety or other anxiety disorders should not be overlooked. A study out of Australia indicates that adults who suffer from stuttering have six- to sevenfold increased odds of having an anxiety disorder, specifically the study indicated a 16- to 34-fold increased odds of meeting criteria for DSM IV or ICD-10 social phobia, fourfold increased odds of meeting criteria for DSM IV generalized anxiety disorder, and sixfold increased odds of meeting criteria for ICD-10 panic disorder (Iverach et al., 2009b). Other studies indicate adults with persistent stuttering report high levels of trait, state, and social anxiety, independent of the severity of stuttering speech, and oftentimes warrant a comorbid diagnosis of social phobia. High anxiety often predicts poor treatment outcomes in standard speech programs. An Australian questionnaire of 300 speech-language pathologists (SLPs) and 300 stuttering adults indicate that 65% of SLPs treating stuttering report utilizing anxiety management strategies despite no formal anxiety management training (Menzies et al., 2008).

Cognitive behavioral therapy is a psychotherapeutic intervention that may be useful for stuttering, especially because of the high co-occurrence of social anxiety or other anxiety disorders. A clinical trial of CBT combined with speech restructuring treatment indicated that while CBT had no impact on stuttering frequency, CBT treatment was associated with less anxiety and avoidance of daily speaking situations (Menzies et al., 2008).

More interventional forms of treatment, as well as different forms of neuromodulation, have also been studied. Recent research has attempted to pair transcranial direct current stimulation (tDCS) to the left inferior frontal cortex, known to be underactive during speaking in those who stutter, in order to improve behavioral therapy interventions including choral speech and metronome-timed speech (Chesters et al., 2018). Daily application of 20 min of 1-mA anodal tDCS over the left inferior frontal cortex combined with tasks performed under choral and metronome-timed speaking conditions for five consecutive days indicated a significant reduction in disfluency at 1 week post-intervention that was maintained in reading tasks at 6 weeks, compared to the same behavioral intervention paired with sham stimulation. However, conversation tasks returned to pre-intervention baseline levels (Chesters et al., 2018).

Repetitive transcranial magnetic stimulation (rTMS) is another form of neuromodulation that alters the brain’s electrical activity with large magnets oriented outside the skull. TMS potentials have been used to reconstruct timed neural integration in intracortical motor networks to further our understanding of functional brain dynamics in people who stutter, with future possibility in clinical treatment (Busan et al., 2019). On the maximally invasive end of the spectrum, deep brain stimulation (DBS) involves the insertion of programmed electrodes into the brain and is FDA approved for the treatment of Parkinson’s disease and essential tremor. There are cases in the literature of DBS treatment for acquired stuttering, and recently the first case was published of DBS treatment for developmental stuttering (Maguire et al., 2012; Lochhead et al., 2016). This DBS case for developmental stuttering has since been replicated in France (Thiriez et al., 2013). A patent has since been filed by Medtronic for the treatment of stuttering by DBS.

## Discussion

The pharmacologic treatment of stuttering has progressed from the earliest dopamine-blocking medications to a variety of second-generation dopamine-blocking medications with more tolerable side-effect profiles. However, even with numerous studies indicating the benefits of pharmacological treatment in reducing the burden of disease, no medications to date have been FDA approved. We postulate that one reason for this discrepancy is that no company has been willing to invest the hundreds of millions of dollars to push medications through the FDA process. While medications have shown benefit in the past, like antipsychotics, all are already generic and have no patent extension; thus, no financial incentive exists to have one of these medications studied and submitted for FDA approval. However, currently there are two active medications, mentioned previously and under patent, ecopipam and deutetrabenazine, that are currently going through clinical trials with the hope of eventually being FDA approved for stuttering.

Future directions include further investigation of these medications, which have a unique activity on dopamine. Another potential therapeutic target for medications is the modification of lysosomal storage, suggesting that further research in this area is needed. Additional research is also needed to address stuttering in adolescents, since some FDA-approved medications do not include research in this population. Future research should also include improving the accuracy of assessing changes in stuttering severity. Although global scales are consistent with treatment effect, stuttering research needs standardization among quantitative measures of outcomes in order to improve comparisons between medications. As for now, we have no head-to-head comparative trials between speech therapy and pharmacologic treatment of stuttering, and with different raters and subject pools, comparative analyses cannot be adequately performed. However, future studies should include three arms in the same randomized sample with the appropriate inter-rater reliability – speech therapy alone, medication alone, and speech therapy combined with medication. One can postulate that stuttering will be similar to other neuropsychiatric conditions such as depression where “talk” therapy combined with medication will be the most effective form of therapy. Moving forward, psychiatrists, due to their knowledge in both psychotherapy and psychotropic medications, should serve an integral part of the treatment team along with phoniatric physicians, speech-language pathologists, and psychologists. Psychiatrists should partner with these specialists in order to optimize the treatment of stuttering.

## Author Contributions

DN, TK, and KS wrote this review article with the mentorship, guidance, editing, and assistance of GM.

## Conflict of Interest

The authors declare that the research was conducted in the absence of any commercial or financial relationships that could be construed as a potential conflict of interest.
